# Characterizing Failure to Establish Hepatitis C Care of Baby Boomers Diagnosed in the Emergency Department

**DOI:** 10.1093/ofid/ofw211

**Published:** 2016-10-04

**Authors:** Ricardo A. Franco, E. Turner Overton, Ashutosh R. Tamhane, Jordan M. Forsythe, Joel B. Rodgers, Julie K. Schexnayder, Deanne Guthrie, Suneetha Thogaripally, Anne Zinski, Michael S. Saag, Michael J. Mugavero, Henry E. Wang, James W. Galbraith

**Affiliations:** ^1^Division of Infectious Diseases,; ^2^Emergency Department,; ^3^VA Quality Scholars Program, and; ^4^Department of Education, University of Alabama School of Medicine, Birmingham, Alabama;; ^5^University of Alabama at Birmingham Health System

**Keywords:** emergency department, hepatitis C screening, linkage to care.

## Abstract

**Background.:**

Emergency departments (EDs) are high-yield sites for hepatitis C virus (HCV) screening, but data regarding linkage to care (LTC) determinants are limited.

**Methods.:**

Between September 2013 and June 2014, 4371 baby boomers unaware of their HCV status presented to the University of Alabama at Birmingham ED and underwent opt-out screening. A linkage coordinator facilitated referrals for positive cases. Demographic data, *International Classification of Diseases, Ninth Revision* codes, and clinic visits were collected, and patients were (retrospectively) followed up until February 2015. Linkage to care was defined as an HCV clinic visit within the hospital system.

**Results.:**

Overall, 332 baby boomers had reactive HCV antibody and detectable plasma ribonucleic acid. The mean age was 57.3 years (standard deviation = 4.8); 70% were male and 61% were African Americans. Substance abuse (37%) and psychiatric diagnoses (30%) were prevalent; 9% were identified with cirrhosis. During a median follow-up of 433 days (interquartile range, 354–500), 117 (35%) linked to care and 48% needed inpatient care. In multivariable analysis, the odds of LTC failure were significantly higher for white males (adjusted odds ratio [aOR], 2.57; 95% confidence interval [CI], 1.03–6.38) and uninsured individuals (aOR, 5.16; 95% CI, 1.43–18.63) and lower for patients with cirrhosis (aOR, 0.36; 95% CI, 0.14–0.92) and access to primary care (aOR, 0.20; 95% CI, 0.10–0.41).

**Conclusions.:**

In this cohort of baby boomers with newly diagnosed HCV in the ED, only 1 in 3 were linked to HCV care. Although awareness of HCV diagnosis remains important, intensive strategies to improve LTC and access to curative therapy for diagnosed individuals are needed.

Therapy advances are shifting treatment paradigms for chronic hepatitis C virus (HCV) infection. The demand for broader access to curative HCV therapy is rapidly growing. To benefit from antiviral therapy, persons with HCV infection must be aware of their infection, have access to HCV care, and be engaged in care [1]. In the United States, it is estimated that 50% of HCV-infected individuals remain unaware of their infection [2]. Furthermore, more than 60% of individuals aware of their HCV infection are not receiving medical care [3]. In order to increase HCV diagnosis, the Centers for Disease Control and Prevention revised screening recommendations to include one-time testing of all baby boomers (persons born between 1945 and 1964), who account for 75% of the infection burden in the United States [4].

Emergency Departments (EDs) have proven to be high-yield settings for detecting HCV infection. We have previously described the high prevalence of unrecognized HCV infection in the ED [5]. Systematic ED screening of baby boomers has revealed HCV-antibody (Ab) prevalence rates between 11.1% and 13.7%, with disproportionately high prevalence among uninsured and underinsured persons [5, 6]. Although this process is in excellent position for high-yield HCV detection, little is known about the rates of linkage to care (LTC) after diagnosis in this setting. In the first 6 weeks of the screening activities in our ED, we reported 102 ribonucleic acid (RNA)-positive cases and recorded call-back attempts to 100 of them. Of those, 54 individuals were successfully contacted by phone within 5 call-back attempts, 38 received a confirmed appointment with an HCV specialist, and 21 attended their initial appointment. This preliminary experience suggested that significant barriers exist for successful LTC. This is in alignment with findings from another study in which approximately 60% of patients diagnosed in the ED did not link to HCV care within 12 months of follow up. The authors of this large retrospective analysis suggested that augmentation of ED testing would need to be reinforced by services to improve LTC. The study was limited by using HCV RNA testing as a proxy for LTC and inability to control for factors such as active injection drug use or mental illness [7].

Based on the above, it becomes important to assess the following: (1) the rates of LTC after HCV screening of baby boomers by expanding our preliminary experience and by measuring LTC based on actual attendance to HCV clinics; and (2) the role of competing medical priorities, lack of access to care, and other demographic variables in preventing or delaying LTC of this population diagnosed in the ED. In order to inform best LTC practices, the purpose of this study is to report on failure rates of linkage to HCV care in a cohort of baby boomers newly diagnosed in the ED at a University Hospital, describe the clinical and demographic characteristics of HCV-infected baby boomers, and determine factors associated with LTC failure.

## METHODS

### Study Design

This is a retrospective cohort study of baby boomers newly diagnosed with HCV infection by means of screening in the ED. We systematically offered HCV testing to baby boomers unaware of their status who presented to the University of Alabama at Birmingham (UAB) ED from September 2013 to June 2014 for routine care.

### Setting

The UAB Hospital is an academic, 1100-bed, tertiary care center serving the greater Alabama region. Procedures of the ED HCV Screening Program are described in detail elsewhere [5]. In brief, our program offered opt-out screening to all medically stable baby boomers presenting to the ED for care. Hepatitis C virus Ab testing (Abbott ARCHITECT) is performed in patients who are (1) unaware of their HCV status, (2) medically stable, and (3) able to verbally answer HCV screening prompts in the electronic health record (EHR) to trained nurses. Results of HCV Ab testing are available in 20 minutes. Reflexive HCV viral load specimen collection occurs in the ED for individuals with a reactive HCV Ab test, and HCV viral load testing (polymerase chain reaction [PCR]) is performed with a turnaround time of 2 days.

Patients were informed of the HCV Ab results during the ED visit, and a trained linkage coordinator called HCV-Ab-reactive individuals by phone to deliver HCV confirmatory PCR test results and to assist with follow-up in primary care and HCV clinics—1917 HIV Clinic, UAB Highlands Infectious Diseases, UAB Highlands Gastroenterology Clinic, Hepatology/Gastroenterology at UAB Kirklin Clinic, and Liver Transplant Clinic. Individuals were also instructed to contact the linkage coordinator during posttest counseling in the ED. Individuals not successfully contacted within 5 call-back attempts were mailed a letter informing of their abnormal laboratory results and contact information of the LTC coordinator. The linkage coordinator also promoted HCV education and awareness, helped with charity care applications, and clarified insurance benefits when applicable. Details of LTC methods are described in detail elsewhere [5].

### Covariates

Electronic Health Record (Cerner, Kansas City, MO) queries of the UAB system provided patient-level demographic information and *International Classification of Diseases, Ninth Revision* (ICD-9) coding entries of ED visits; ICD-9 codes for each patient were visit-specific, based on formatted financial numbers. Demographic information included age (at HCV testing), sex, race, marital status, health insurance, and complete address. Only 4 HCV-infected baby boomers were self-identified Hispanics, and we excluded them from analysis. The study cohort generated 1631 unique ICD-9 coding entries. After this list of unique codes was generated, 2 observers independently selected and categorized 500 codes representing medical conditions of interest. This selection occurred after querying the database and was based on conditions commonly diagnosed in the ED and perceived by the investigators as potential competing priorities to LTC. This selection was also based on published methods to assess the prevalence of multimorbidity in large datasets, either in primary care setting or in the general population, described elsewhere [8]. To adapt to the ED setting, we included both acute and chronic medical conditions to allow for inclusion of frequent acute conditions that otherwise are not taken into account in validated methods to assess prevalence and severity of multimorbidity in primary care. We categorized conditions within 18 morbidity domains: Arrhythmia, Back and Neck Disease, Cancer, Chronic Obstructive Pulmonary Disease/Asthma, Chronic Pain, Cirrhosis, Coronary Artery Disease, Diabetes Mellitus, Heart Failure, Hypertension, Kidney Disease, Obesity, Peripheral Vascular Disease, Psychiatric Disease, Rheumatologic Disease, Stroke, Substance Abuse, and Thromboembolic Disease. Disagreement between observers was solved by further discussions about whether the ICD-9 code in question represented a matching condition of interest. Accordingly, medical conditions deemed by both observers not to be a match to the determined morbidity domains were excluded. We also excluded all ICD-9 codes of injuries and surgical conditions. We queried the EHR for visits to HCV clinics (1917 HIV Clinic, UAB Highlands Infectious Diseases, UAB Highlands Gastroenterology Clinic, Hepatology/Gastroenterology at UAB Kirklin Clinic, and Liver Transplant Clinic); the ED would refer directly to 2 clinics (UAB Liver Center and 1917 Clinic), but we also queried 2 other clinics that were also points of HCV care and self-referrals (UAB Highlands Gastro and UAB Highlands Infectious Diseases). We also queried inpatient visits, outpatient visits to Primary Care (including primary care and Internal Medicine ambulatory services available on campus and in satellite clinics that are part of the UAB Health System around Birmingham City area), and other medical specialties (Anticoagulation Clinic, Audiology, Cardiology, Dermatology, Endocrinology, Hematology/Oncology Nephrology, Neurology, Nutrition, Obstetrics/Gynecology, Occupational Medicine, Ophtalmology, Orthotics, Otolaringology, Psychiatry, Pulmonary, Radiology, Social Services, Surgery [General and Subspecialties], Rehabilitation, Rheumatology, Urgent Care, Urology) that occurred after HCV screening in the ED and throughout the study follow-up period. The UAB Institutional Review Board approved this analysis of testing and LTC program and accessing EHR.

### Statistical Analysis

Initial exploration of the data began with descriptive statistics of various continuous and categorical variables. Continuous variables were reported using mean and standard deviation or median and quartiles depending upon normal/skewed distribution, respectively. Categorical variables were reported as frequencies and percentages. The primary outcome was a dichotomous measure (“Show” versus “NoShow”) of whether HCV-infected baby boomers attended at least 1 HCV clinic visit after diagnosis in the ED. Factors associated with LTC failure (NoShow) were examined using univariate and multivariable logistic regression modeling and reported as (crude) odds ratios (ORs) and adjusted ORs (aOR), respectively, with 95% confidence intervals (CIs). In multivariable analyses, the clinically relevant factors were included as described previously. Model performance was examined using c-statistics, max-rescaled r-square, and Hosmer-Lemeshow test for model fit. Multicollinearity of the independent factors was examined with variance inflation factor (VIF) by adjusting the linear combinations by the weight matrix used in the maximum likelihood algorithm [9]; the VIF for all the factors was <3.0, indicating no multicollinearity. Statistical significance was set at 0.05 (2-tailed). Statistical analyses were performed using SAS statistical software, version 9.3 (SAS Institute, Cary, NC).

## RESULTS

Overall, 4371 baby boomers were screened from September 2013 to June 2014, 473 of whom tested positive for HCV Ab (11%). Not all patients had HCV VL test performed; patients either left the ED before extra blood collection or did not have extra blood work performed as part of their ED diagnostics (lack of enough blood volume or coagulated sample). Hepatitis C viral load could be confirmed in 402 patients (85%) and HCV viremia was detected in 332 (83%). These 332 patients were then (retrospectively) followed until February 2015 allowing follow-up of 8 to 17 months.

**Figure 1.**
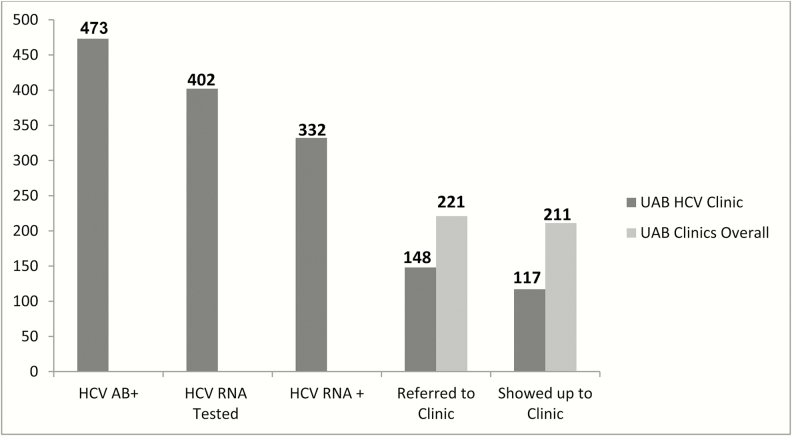
Hepatitis C virus (HCV) care cascade of baby boomers newly diagnosed with chronic hepatitis C in the University of Alabama at Birmingham, Birmingham, Alabama (UAB) emergency department (ED) screened from Septemeber 2013 to June 2014, examining linkage to HCV clinics and utilization of other medical and surgical clinics within the UAB Health System. Nearly two thirds of HCV-infected baby boomers used the healthcare system for medical or surgical outpatient visits after HCV diagnosis in ED, but only 1 of 3 patients attended HCV care during follow up. AB, antibody; RNA, ribonucleic acid.

Among the 332 HCV-viremic baby boomers included, 221 (66%) patients were referred and notified of appointments to any outpatient care within campus and 211 (64%) used any outpatient care within campus. In regards to HCV clinics only, 148 patients were notified of appointments and 117 (35%) attended HCV clinics at least once during a median follow-up of 433 days (interquartile range [IQR], 354–500), as shown in Figure 1. Overall, 121 baby boomers (36%) failed to attend any clinic visits after HCV screening, and 94 (28%) attended non-HCV care visits only. The median time between diagnosis and the first HCV clinic visit was 81 days (IQR, 40–205 days). The mean age at the time of screening was 57.3 years: 70% were males and 61% were African Americans (AAs). Twenty-seven percent were Medicaid beneficiaries and 14% were uninsured. The majority (77%) of individuals lived in low income zip codes, and only 17% reported married status. Most clinic attendees (95%) lived in urban areas and were residents of Birmingham City (70%). In univariate analysis, nonmarried status and lack of insurance were significantly associated with nonattendance to HCV clinics (Table 1).

**Table 1.** Sociodemographic and Economic Characteristics and Their Association With Linkage to Care Failure^a^ in Baby Boomers Newly Diagnosed With Chronic Hepatitis C in the UAB ED Screening From Septemeber 2013 to June 2014

Characteristic	All N = 332 N (%)	“NoShow”^a^ N = 215 n (%)	“Show”^a^ N = 117 n (%)	OR^b^ (95% CI)^b^	*P* Value^c^
Aged (years), mean ± SD	57.3 ± 4.8	57.4 ± 5.0	57.0 ± 4.6	1.02^d^ (0.97–1.06)	.53
Race, sex					
African American					
Male	143 (43)	91 (42)	52 (44)	1.22 (0.59–2.51)	.59
Female	59 (18)	34 (16)	25 (21)	0.95 (0.42–2.15)	.89
White					
Male	91 (27)	67 (31)	24 (21)	1.94 (0.88–4.28)	.10
Female	39 (12)	23 (11)	16 (14)	Ref	
Marital Status					
Not married^e^	276 (83)	186 (87)	88 (75)	2.27 (1.27–4.06)	.006
Married	56 (17)	27 (13)	29 (25)	Ref	
Health insurance					
Uninsured	46 (14)	43 (20)	3 (3)	9.50 (2.82–32.04)	<.001
Public^f^	138 (42)	83 (39)	55 (47)	1.00 (0.62–1.61)	1.00
Private	148 (44)	89 (41)	59 (50)	Ref	
Residence^g^					
Nonlocal county	58 (18)	38 (18)	20 (17)	0.95 (0.52–1.74)	.87
Local county	40 (12)	21 (10)	19 (16)	0.55 (0.28–1.09)	.09
Birmingham city	233 (70)	155 (72)	78 (67)	Ref	
Low income zip code^h^					
Yes	252 (77)	163 (77)	89 (76)	1.07 (0.63–1.82)	.81
No	76 (23)	48 (23)	28 (24)	Ref	
Rural county^i^					
Yes	16 (5)	13 (6)	3 (3)	2.46 (0.69–8.81)	.17
No	315 (95)	201 (94)	114 (97)	Ref	

Abbreviations: ED, emergency department; HCV, hepatitis C virus; OR, odds ratio; Ref, reference category; SD, standard deviation; UAB, University of Alabama at Birmingham, Birmingham, Alabama.

^a^Whether attended (Show) or not attended (NoShow, linkage to care failure) at least 1 HCV clinic visit after HCV diagnosis in the ED.

^b^Univariate logistic regression.

^c^Wald χ^2^.

^d^Per 1-year increase in age (at the time of screening).

^e^Not married (N = 274) included single (n = 173), divorced (n = 62), separated (n = 20), widowed (n = 19), and unknown (n = 2).

^f^Public insurance (N = 138) included Medicaid (n = 88) and Medicare (n = 50).

^g^Local county = Jefferson County.

^h^Low income zip code: median household income less than or equal to $32000. US Census Bureau for Census 2010.

^i^Rural County: based on “List of Rural Counties And Designated Eligible Census Tracts in Metropolitan Counties”: Updated Census 2010.

In this cohort, 84% of patients had multimorbidity (defined as having ≥2 of the morbidity domains), 35% had ≥5 morbidity domains, and 48% were hospitalized at least once during the study period (Table 2). Highly prevalent domains included Substance Abuse (37%), Psychiatric Disease (30%), and Cirrhosis (9%). On univariate analysis, those with Substance Abuse (OR, 1.91; 95% CI, 1.17–3.11; *P* = .01), <5 morbidities (OR, 1.74; 95% CI, 1.09–2.78; *P* = .02) were significantly at greater odds of LTC failure. Whereas those with cirrhosis, chronic kidney disease, diabetes mellitus, attendance to clinics (specialty and primary care), and hospitalization were at significantly lower odds of LTC failure (Table 2).

**Table 2.** Clinical Characteristics and Their Association With Linkage to Care Failure^a^ in Baby Boomers Newly Diagnosed With Chronic Hepatitis C in the UAB ED Screened From Septemeber 2013 to June 2014

Characteristic	All Patients N = 332 N (%)	NoShow^a^ N = 215 n (%)	Show^a^ N = 117 n (%)	OR^b^ (95% CI)^b^	*P* Value^c^
Substance Abuse					
Yes	122 (37)	90 (42)	32 (27)	1.91 (1.17–3.11)	.01
No	210 (63)	125 (58)	85 (73)	Ref	
Psychiatric Disease					
Yes	98 (30)	70 (33)	28 (24)	1.53 (0.92–2.56)	.10
No	234 (70)	145 (67)	89 (76)	Ref	
Cirrhosis					
Yes	31 (9)	14 (7)	17 (15)	0.41 (0.19–0.87)	.02
No	301 (91)	201 (93)	100 (85)	Ref	
CAD					
Yes	58 (17)	41 (19)	17 (15)	1.39 (0.75–2.57)	.30
No	274 (83)	174 (81)	100 (85)	Ref	
CHF					
Yes	50 (15)	28 (13)	22 (19)	0.65 (0.35–1.19)	.16
No	282 (85)	187 (87)	95 (81)	Ref	
Hypertension					
Yes	175 (53)	113 (53)	62 (53)	0.98 (0.63–1.54)	.94
No	157 (47)	102 (47)	55 (47)	Ref	
Stroke					
Yes	35 (11)	24 (11)	11 (9)	1.21 (0.57–2.57)	.62
No	297 (89)	191 (89)	106 (91)	Ref	
Cancer					
Yes	39 (12)	28 (13)	11 (9)	1.44 (0.69–3.02)	.33
No	293 (88)	187 (87)	106 (91)	Ref	
CKD					
Yes	44 (13)	21 (10)	23 (20)	0.44 (0.23–0.84)	.01
No	288 (87)	194 (90)	94 (80)	Ref	
COPD/Asthma					
Yes	69 (21)	44 (20)	25 (21)	0.95 (0.55–1.65)	.85
No	263 (79)	171 (80)	92 (79)	Ref	
Diabetes Mellitus					
Yes	87 (26)	45 (21)	42 (36)	0.47 (0.29–0.78)	.003
No	245 (74)	170 (79)	75 (64)	Ref	
Morbidity Count					
0 or 1	52 (16)	32 (15)	20 (17)	1.24 (0.63–2.41)	.53
2 to 4	163 (49)	117 (54)	46 (39)	1.97 (1.19–3.24)	.01
≥5	117 (35)	66 (31)	51 (44)	Ref	
Primary Care					
Yes	60 (18)	17 (8)	43 (37)	0.15 (0.08–0.28)	<.001
No	272 (82)	198 (92)	74 (63)	Ref	
Specialty Visit					
Yes	231 (70)	101 (47)	117 (100)	0.01 (0.00–0.03)^d^	<.001
No	101 (30)	114 (53)	0 (0)	Ref	
Surgery Visit					
Yes	109 (33)	53 (25)	56 (48)	0.36 (0.22–0.57)	<.001
No	223 (67)	162 (75)	61 (52)	Ref	
Hospitalization					
Yes	158 (48)	84 (39)	74 (63)	0.37 (0.23–0.59)	<.001
No	174 (52)	131 (61)	43 (37)	Ref	

Note: Results not displayed for “Arrhythmia”, “Chronic Back and Neck Disease”, “Chronic Pain”, “Obesity”, “Rheumatologic Disease”, “Venous Thromboembolic Disease”. These domains resulted in statistically nonsignificant associations with attendance to HCV clinic visits. These domains were included in “Morbidity Counts”.

Abbreviations: CAD, coronary artery disease; CHF, congestive heart failure; CKD, chronic kidney disease; COPD, chronic obstructive pulmonary disease; ED, emergency department; HCV, hepatitis C virus; OR, odds ratio; Ref, reference category; SD, standard deviation; UAB, University of Alabama at Birmingham, Birmingham, Alabama.

^a^Whether attended (Show) or not attended (NoShow, linkage to care failure) at least 1 HCV clinic visit after HCV diagnosis in the ED.

^b^Univariate logistic regression.

^c^Wald χ^2^.

^d^”Exact” logistic regression.

On multivariable analysis (Table 3), white males (aOR, 2.57; 95%, CI 1.03–6.38) and uninsured status (aOR, 5.16; 95% CI, 1.43–18.63) were strongly and significantly associated with LTC failure. Cirrhosis (aOR, 0.36; 95%, CI 0.14–0.92), primary care visit within the university system (aOR, 0.20; 95% CI, 0.10–0.41), and hospitalization (aOR, 0.55; 95% CI, 0.31–0.99) were at lower odds of LTC failure. In stratified analysis limited to patients with insurance (private and public), the strength of these associations remained strong, although not statistically significant due to smaller sample sizes in each stratified group.

**Table 3.** Multivariable Analysis^a^ Examining Association of Sociodemographic, Economic, and Clinical Characteristics With Linkage to Care Failure^b^ in Baby Boomers Newly Diagnosed With Chronic Hepatitis C in the UAB ED Screened From Septemeber 2013 to June 2014

Characteristic	Adjusted^a^ OR (95% CI)	*P* Value^c^
Race-sex		
African American Male	1.86 (0.75–4.64)	.18
African American Female	1.28 (0.45–3.59)	.65
White Male	2.57 (1.03–6.38)	.04
White Female	Ref	—
Marital status		
Not Married^d^	1.71 (0.86–3.40)	.13
Married	Ref	—
Health insurance^e^		
Uninsured	5.16 (1.43–18.63)	.01
Public Insurance	0.97 (0.55–1.70)	.91
Private	Ref	—
Residence^f^		
Nonlocal county	0.80 (0.28–2.26)	.67
Local county	0.62 (0.23–1.67)	.34
Birmingham city	Ref	—
Low income zip code^g^		
Yes	0.76 (0.30–1.97)	.58
No	Ref	—
Rural county^h^		
Yes	2.27 (0.48–10.83)	.30
No	Ref	—
Substance Abuse		
Yes	1.67 (0.93–3.02)	.09
No	Ref	—
Psychiatric Disease		
Yes	1.35 (0.72–2.54)	.35
No	Ref	—
Morbidity Count		
<5	0.99 (0.55–1.86)	.97
≥5	Ref	—
Cirrhosis		
Yes	0.36 (0.14–0.92)	.03
No	Ref	—
Hospitalization		
Yes	0.55 (0.31–1.00)	.05
No	Ref	—
Primary Care Visit		
Yes	0.20 (0.10–0.41)	<.001
No	Ref	—
Surgery Clinic Visit		
Yes	0.59 (0.33–1.05)	.07
No	Ref	—

Abbreviations: CI, confidence interval; ED, emergency department; HCV, hepatitis C virus; OR, odds ratio; Ref, reference category; UAB, University of Alabama at Birmingham, Birmingham, Alabama.

^a^Multivariable logistic regression modeling (Hosmer-Lemeshow test *P* value, .25; C-statistics, 79.7%; Max-rescaled R-square, 33.1%).

^b^Whether attended (Show) or not attended (NoShow, linkage to care failure) at least 1 HCV clinic visit after HCV diagnosis in the ED.

^c^Wald χ^2^.

^d^Not married (N = 274) included single, divorced, separated, widowed, and unknown. Unknown (n = 2).

^e^Public insurance (N = 138) included Medicaid (n = 88) and Medicare (n = 50).

^f^Local county = Jefferson County.

^g^Low income zip code: median household income less than or equal to $32000. US Census Bureau for Census 2010.

^h^Rural County: based on “List of Rural Counties And Designated Eligible Census Tracts in Metropolitan Counties”: Updated Census 2010.

## DISCUSSION

In this study, only 1 of 3 baby boomers with newly diagnosed HCV infection were successfully LTC in HCV clinics. Here, we characterized the “no show phenomenon” to HCV care and the potential role played by demographic and clinical factors, similarly to what has been described in human immunodeficiency virus care [10]. Our cohort was largely composed of vulnerable minorities with frequent comorbid conditions. Surrogates of access to care (attendance to specialty or primary care, and insurance) were highly associated with successful LTC. The majority of patients with positive screening did not link to care in our referral system. This finding is consistent with a recent report by White et al [11], in which 23% of individuals (baby boomers and intravenous drug users) successfully attended HCV care visit within 6 months of ED diagnosis. In our cohort, 25% of patients who achieved LTC (83 of 332) did so in the first 6 months of follow up.

The cohort of HCV-viremic baby boomers was mostly comprised by aging, nonmarried, AA males living in inner-city, low income areas. Male gender (especially whites in this study) was an independent predictor associated with lack of attendance to HCV clinics. Male gender is associated with lack of healthcare utilization; men are more likely to engage in detrimental lifestyle (such as smoking and alcohol abuse), have lower perceptions of disease risk, and be unwilling to commit to preventive care [12–14]. Despite accepting HCV opt-out testing and counseling in the ED, individuals may have postponed engagement in HCV care in favor of competing health priorities and/or competing demands such as food security, unstable housing, other financial constraints, and/or lack of transportation [15]. Additional research is warranted to identify specific barriers to healthcare utilization concerning these factors.

The uninsured are less likely to consume preventive care, and the majority of Health System-owned sites in the United States may not offer free primary care for uninsured patients [16–18]. Likewise, our university-based healthcare system allows for privately insured and Medicare patients to attend primary care on campus. Uninsured and Medicaid patients have access to specialty care (HCV clinics included) on campus after securing primary care in our affiliated county hospital, in Federally Qualified Health Centers, or in the clinics affiliated with the Jefferson County Department of Health. Uninsured patients must also be approved by the UAB Hospital’s Charity Care Program before receiving an appointment at UAB Hospital or Affiliated Clinics. Despite being a Disproportionate Share Hospital, the inability of the UAB Health System to cover primary care for uninsured and Medicaid patients on campus was perceived as a challenge to patients and to our linkage program. Among a broad array of demographic and clinical factors included in our model, lack of insurance was the strongest independent predictor of LTC failure. These results are consistent with a recent report by Linas et al [7]. In their large, retrospective analysis, diagnosis in outpatient clinics, private insurance, and engagement in care (≥10 visits) were all independent predictors of successful LTC.

In addition to the above-mentioned data, the high yield of HCV testing and the large amount of newly diagnosed patients far exceeded expectations and resources available for LTC of vulnerable individuals. The linkage coordinator promoted education and awareness, referrals to primary care, helped with charity care applications when applicable, and clarified insurance benefits. The HCV linkage program was funded to mainly cover the costs of HCV antibody and RNA testing (such testing is not covered by insurance, neither by charity care funds in the ED setting) and to cover the efforts of the linkage coordinator. Under limited resources and the high volume of seropositive patients diagnosed in a short period of time, there were not enough resources to help patients filing for insurance or help them with transportation and other barriers.

African Americans are disproportionally affected by HCV with higher prevalence of infection, higher liver-related morbidity and mortality, and underrepresentation in cohorts undergoing HCV treatment compared with whites [19]. When accounting for both men and women, there were no racial disparities in LTC among AAs and whites in our study. It is interesting to note that AAs had slightly higher prevalence of HCV clinic attendance compared with whites (38% vs 31%), albeit not statistically significant (OR, 0.72; 95% CI, 0.45–1.15). We hypothesize that our ED screening program expanded HCV care access to inner-city minorities. African Americans may have had preferential use of HCV care on campus compared with whites who predominantly live in the suburbs. However, our analysis was limited to the university healthcare system, not capturing utilization of other clinics in the vicinity of Birmingham City.

Multimorbidity, defined here as the presence of ≥2 morbidity domains, was present in 84% of our cohort. We also verified that approximately half of these patients required at least 1 hospitalization after HCV diagnosis, which alludes to high morbidity burden and potential barriers to effective LTC. However, our study did not detect independent associations between several morbidity domains and LTC failure. In fact, morbidity domains such as Cirrhosis, Chronic Kidney Disease, and Diabetes were LTC-enabling factors. Cirrhosis did maintain strong and independent association with lower odds of LTC failure in adjusted analysis. The root reasons for these associations are unclear. We hypothesize that patients with these conditions may have higher perceptions of disease risk (and/or poorer quality of life) and remain engaged in care long term, enabling attendance to HCV clinics. Nevertheless, these same enabling conditions could potentially prevent expeditious LTC and HCV treatment uptake (especially if not under optimal control), and further studies focused on time to linkage outcomes are warranted.

The HCV care cascade sets the foundation to identify gaps in the continuum of care and evaluate the impact of public health interventions [3]. However, this model is limited to assessments at single time points of the care cascade, not accounting for the longitudinal nature of the care continuum [20]. By assessing linkage to HCV clinics longitudinally, the median time interval between screening and HCV clinic attendance neared 3 months (81 days; IQR, 40–205). Similar delays had been observed by White et al [8] in their early experience with integrated HCV screening in the ED and LTC (97 days; IQR, 49–153). These observations may provide useful parameters for future planning and evaluation of LTC interventions based on time to endpoints and be helpful in setting realistic goals for these programs.

Our study had several limitations. We could only track LTC outcomes within our healthcare system, not capturing visits that may have occurred in outside centers. However, we estimate uptake by outside clinics to be low, given the barriers of access to care and competing medical priorities as described above. Furthermore, our analysis relied on EHR queries of clinical data that is tailored to routine patient care in ED and biased towards underreporting of ICD-9 codes. This limitation may have underestimated the frequencies of competing medical priorities, but it is advantageous in describing active medical issues present at the time of HCV diagnosis in the ED. Study limitations are also related to our relatively small sample size, short-term follow up, and lack of validated methods to assess the prevalence of multimorbidity in large datasets generated by medical care in ED setting.

## CONCLUSIONS

Approximately 1 of every 3 HCV-infected baby boomers was linked to HCV care after ED screening. Lack of health insurance was the strongest independent predictor of LTC failure after HCV screening. Linkage to care interventions in this setting should be robust to address significant lack of access to care, frequent competing priorities, and meet the need of high-yield screening strategies. Further research is warranted for optimal linkage to HCV care practices in ED settings.
